# First Report of SNPs Detection in TMEM154 Gene in Sheep from Poland and Their Association with SRLV Infection Status

**DOI:** 10.3390/pathogens14010016

**Published:** 2024-12-30

**Authors:** Magdalena Materniak-Kornas, Katarzyna Piórkowska, Katarzyna Ropka-Molik, Adrianna Dominika Musiał, Joanna Kowalik, Anna Kycko, Jacek Kuźmak

**Affiliations:** 1 Department of Biochemistry, National Veterinary Research Institute, 24-100 Pulawy, Poland; 2 Department of Animal Molecular Biology, National Research Institute of Animal Production, 32-083 Balice, Polandadrianna.musial@iz.edu.pl (A.D.M.); 3 Department of Pathology, National Veterinary Research Institute, 24-100 Pulawy, Poland

**Keywords:** small ruminant lentiviruses (SRLVs), sheep, genotyping, TMEM154, single nucleotide polymorphism

## Abstract

Small ruminant lentiviruses (SRLVs) infect sheep, causing a multiorganic disease called maedi-visna or ovine progressive pneumonia, which significantly affects the production and welfare of sheep, generating serious economic losses. Although not all infected animals develop fully symptomatic disease, they constantly spread the virus in the flock. Since the infection is incurable and no vaccine is available, another approach is necessary to control SRLV infections. In recent years, an alternative for culling infected animals has become the approach based on identifying genetic markers for selecting SRLV-resistant individuals. Recent reports revealed several candidates, including gene encoding transmembrane protein 154 (TMEM154). Several single nucleotide polymorphisms (SNPs) are found within this gene in sheep of different breeds, but only some can be considered as resistant markers. This study aimed to investigate the presence of single polymorphic sites in TMEM154 gene in sheep of selected Polish flocks and assess their association with the infection and proviral load in the context of susceptibility to SRLV infection. In total 107 sheep, representing three breeds, were screened for their SRLV infection status by serological and PCR testing. All these animals were also genotyped to characterize the presence of SNPs in TMEM154 gene and estimate their potential of being the SRLV-resistance marker. The frequency of identified alleles differed among breeds. Moreover, the positive association between TMEM154 genotype and SRLV status was found for E35K polymorphism and two polymorphic sites in 5′UTR in one of analyzed breed. However, when the relationship between SNPs and SRLV proviral load was analyzed, five had a strong association, considering the whole population of tested sheep. Presented data, for the first time, identified the presence of SNPs in TMEM154 gene in sheep housed in Polish flocks and suggested that selecting SRLV-resistant animals based on this analysis might be possible, but further validation in a larger group of sheep is required.

## 1. Introduction

Small ruminant lentiviruses (SRLVs) are heterogeneous viruses from the Retroviridae family, infecting sheep and goats. Among several genotypes of SRLV that have been identified, the most common are genotype A, including strains closely related to maedi-visna virus (MVV), and genotype B, referred as caprine arthritis-encephalitis virus (CEAV)-like [1]. SRLVs infect primarily monocytes/macrophages of sheep and goats. Infected sheep show an initial viremia, but the virus is cell-associated with proviral DNA, and after that, the infection becomes persistent with restricted replication. Infected monocytes circulate through the organism and infiltrate target organs, including the lung, mammary gland, and synovial membrane of joints. The infection becomes latent and seroconversion typically appears after several months or later. The maturation of monocytes into macrophages triggers virus replication, which slowly activates the immune response, causing chronic inflammatory lesions. At that time, the infected animal is asymptomatic but becomes a persistent source of virus for horizontal transmission through inhalation of respiratory secretions [2]. This is a critical route of SRLV transmission, especially during sheep housing. The other one is the consumption of colostrum and milk from infected dams [3,4], while intrauterine transmission is a possible but sporadic way of infection [5]. The persistent chronic SRLV infection leads to a progressive wasting disease called maedi-visna (in the US known as ovine progressive pneumonia (OPP)) in about one-third of infected sheep. At the same time, a similar percentage of goats causes emaciation, progressive arthritis, and mastitis [2]. SRLV infections in sheep are spread worldwide, including Poland, with a seroprevalence of 33.3% [6]. Subclinical infections predispose animals to the diseases that decrease fertility, resulting in reduced offspring production. Unfortunately, there is no vaccine or effective treatment against SRLV infection, and the only way to prevent the spreading of the virus seems to be through control programs, which have already successfully been introduced in several countries. The culling of seropositive animals has been reported as the most effective practice. Still, in herds with high infection rates, an alternative approach has been implemented based on the selection of the offspring of seronegative dams and its artificial feeding [4,7]. However, sometimes, it is impossible due to a low number of seronegative ewes, which may lead to the loss of selected genetic traits of the flock; then, the lambs are separated at birth independently of mother’s serological status and reared with virus-free colostrum. Both variants are expensive and time-consuming, as well as complicated due to the prolonged seroconversion and the high genetic variability, which limit the usefulness of diagnostic methods [7,8]. In recent years, there has been a perception that new strategies based on more effective control measures are required. Therefore, many studies have focused on the host factors that may determine the resistance of individual animals or breeds to SRLV infection. The idea is based on the knowledge that some breeds are more susceptible to SRLV infection (Texel, Finnsheep, East Friesian Milk), while others are relatively resistant (Suffolk, Rambouliet, Merinoland) [9,10,11,12,13]. Over ten years ago, Heaton and co-workers [14] reported the discovery of single nucleotide polymorphism (SNP) in an ovine gene associated with SRLV infection in a large multi-breed sheep flock in the U.S. Out of several polymorphisms found in TMEM154 gene encoding transmembrane protein 154, one located in exon 2 (rs408593969 c.103 G>A, missense variant (E35K)) was proposed as the most promising SRLV infection risk marker. The wild-type variant G was suggested to predispose sheep to SRLV infection. In contrast, alternate variant A, responsible for the change of glutamate (E) into lysine (K), was associated with 69-fold reduced susceptibility to infection in alternate homozygous. Although TMEM154 gene was found in most animal species, the function of the encoded protein is still unknown. However, in humans, TMEM154 transcription was found to be upregulated in B cells and monocytes [14], as well as in the context of asthma severity [15], which may suggest its role in the respiratory system. Heaton et al. [16] went further. They implemented their results for the development of standardized genotyping method and its commercialization for the selection of sheep carrying this marker as a new strategy to reduce the susceptibility to SRLV infection. Later, several studies on different breeds of sheep in several countries also suggested the association of E35K polymorphism with susceptibility to SRLV infection, but not in all tested breeds [17,18,19,20]. The association of other SNPs described by Heaton and co-workers [14] in TMEM154 gene with susceptibility to SRLV infection is still disputable since they are either very rarely identified or the results of different studies are discordant [21,22,23,24].

Genotyping of sheep to screen for putative genetic markers of susceptibility has never been performed in sheep from Polish flocks. Therefore, the present study aimed to detect polymorphisms, evaluate the allelic frequencies of TMEM154 gene variations in sheep from three breeds and test their association with SRLV infection status.

## 2. Materials and Methods

### 2.1. Animals and Blood Samples

A total 107 sheep were housed in three flocks located in Southern Poland, each representing a different breed: 52 Polish Mountain sheep (ewes), 37 Olkuska sheep (35 ewes and 2 rams), and 18 Cameroon sheep (ewes). In the years 2021–2023, during control visits to these flocks, local veterinarians found 8 individuals showing clinical symptoms of ovine progressive pneumonia (OPP) (maedi-visna MV), like pneumonia with accompanying dyspnea and cachexia. Furthermore, during these examinations, two animals died, and the corresponding samples were taken and sent to our laboratory for MV confirmation. Lung tissue samples were collected from both of them and fixed in 10% neutral-buffered formalin for histopathology and immunohistochemistry. Blood samples were collected from all sheep by jugular venipuncture into tubes with EDTA (for DNA isolation) and without EDTA (for serological testing) by veterinarians during routine visits. Serum samples were obtained by centrifugation of blood samples at 2600× *g* rpm. Genomic DNA was isolated from peripheral blood leukocytes using the Nucleospin Blood Quick Pure kit (Machery-Nagel, Düren, Germany) and using the Nucleospin Tissue kit (Machery-Nagel, Düren, Germany) according to the manufacturer’s recommendations.

### 2.2. Determination of SRLV Status

The SRLV infection status of sheep was determined based on the serological and PCR tests.

According to the manufacturer’s recommendations, the serological status was confirmed by an enzyme-linked Immunosorbent assay (ELISA) (ID Screen MVV/CAEV Indirect Screening test, IDVet, Grabels, France). Based on that, samples were considered serologically positive with an S/*p* value of ≥ 60% and negative with an S/*p* value of ≤ 50%. No doubtful results (the range between 50% and 60%) were recorded in this study.

SRLV genetic material was detected using nested PCR, as described elsewhere [25]. Briefly, the 625 bp *gag* gene fragment containing the capsid coding sequences was amplified using the following primer pairs: GAGf1 and P15 in the first round and CAGAG5 and CAGAG3 in the second round. The resulting amplicons were analyzed on 1% agarose gels containing ethidium bromide (1 μg/mL) in 1xTAE buffer, cloned into the pCR4.1-TOPO vector (Invitrogen, Waltham, MA, USA) and sequenced from both sides according to the method of Sanger [26] by Genomed, Warsaw, Poland. An animal was considered as infected when ELISA or PCR tests showed a positive result.

### 2.3. SRLV Genotyping

In order to determine SRLV genotypes, bioinformatic analyses was performed based on partial *gag* gene sequences, aligned to reference SRLV sequences, available in GenBank, using the Geneious alignment module within Geneious Pro 5.3 software (Biomatters Ltd., Auckland, New Zealand). The alignment was submitted to the MEGA 6 version [27] for the best model selection measured by the Bayesian information criterion (BIC) and the corrected Akaike information criterion (AICc). According to the Tamura 3-parameter substitution model results using gamma distribution (+G) with five rate categories [28], they were applied in MEGA 6 to infer a phylogenetic tree using the maximum likelihood method. The statistical confidence limits of the phylogram topologies were assessed with 1000 bootstrap replicates [29].

### 2.4. Quantification of SRLV Proviral DNA

Proviral DNA of SRLV was quantified by qPCR assay using Rotor-Gene Q (Qiagen, Hilden, Germany), with primers and probes specific for detected SRLV subtypes, whose presence has been previously confirmed in particular flocks. The Olkuska and Cameroon breed samples were tested using probe-based qPCR assay, and the Polish Mountain breed was tested using an intercalating dye (sybr green). The primers and probes used in the qPCR reaction are shown in Table S1. The standard curve was determined using 10-fold serial dilutions (from 10^6^ to 10 copies) of plasmid DNA containing a 625 bp *gag* gene fragment of specific SRLV detected in a particular flock as the template. Amplification with DNA from Olkuska and Cameroon sheep was carried out in a total volume of 20 µL under the following conditions: preincubation and polymerase activation at 95 °C for 15 min, followed by 45 cycles of 94 °C for 60 s and 60 °C for 60 s. The reaction mixture contained 10 µL of 2× QuantiTect Probe PCR buffer (Qiagen, Hilden, Germany), 400 nM of each primer, 200 nM of a specific probe, and 500 ng of DNA template. Samples from the Polish Mountain breed were tested using a QuantiTect Sybr Green PCR kit (Qiagen, Hilden, Germany)with a total volume of 25 µL; the reaction mixture contained 12.5 µL of 2× QuantiTect Sybr Green PCR buffer, 300 nM of each primer, and 500 ng of DNA template. The following temperature conditions were applied: preincubation and polymerase activation at 95 °C for 15 min, followed by 45 amplification cycles of 94 °C for 15 s, 56 °C for 30 s and 72 °C for 30 s. The analysis of the melting temperature of amplified products confirmed assay specificity. All samples were tested in duplicate, and the results were quantified as the average number of copies per 500 ng of genomic DNA of each sheep.

### 2.5. SNP Identification and Genotyping

The two fragments of TMEM154 spanned exons 1 and 2 and were analyzed in terms of polymorphism identification. The Sanger sequencing method was applied to genotype the previously detected polymorphisms and identify the novel variants. Both amplicons, corresponding to exons 1 and 2, were generated for each analyzed sheep using AmpliTaq Gold™ 360 Master Mix (Thermo Fisher Scientific, Waltham, MA, USA) and primers according to Arcangeli et al. [22] (Table 1). Next, according to the protocol, samples were purified using an enzyme mixture—EPPiC (A&A Biotechnology, Gdynia, Poland). The sequencing using the Sanger method was performed using BigDye™ Terminator v3.1 Cycle Sequencing Kit (Thermo Fisher Scientific) and BigDye XTerminator™ Purification Kit (Thermo Fisher Scientific) according to the manufacturer’s protocol on 3500xL Genetic Analyzer (Applied Biosystems, Thermo Fisher Scientific, Waltham, MA, USA). The Data Collection Software (Applied Biosystems, Thermo Fisher Scientific, Waltham, MA, USA) was applied to raw data analysis.

### 2.6. Histopathology and Immunohistochemistry

The formalin-fixed lung tissue samples were routinely processed, embedded in paraffin and cut on a microtome into 4 μm-thick slides.

For histopathology, the slides were routinely stained with hematoxylin and eosin (HE) and then analyzed under a light microscope (Axiolab, Zeiss, Göttingen, Germany) for the presence of histopathological changes. For immunohistochemistry, the lung tissue sections from the studied sheep and an archival lung tissue section from a sheep that was considered negative were used. The slides were deparaffinized, rehydrated in descending concentrations of ethanol, incubated in 3% H_2_O_2_ diluted in methanol for 10 min to block endogenous peroxidase activity and submitted to antigen retrieval by incubation in a citrate buffer (pH 6.0) for 20 min in a pressure cooker. The slides were then incubated with CAEV5A1 antibody (VMRD, Pullman, WA, USA) diluted at 1:50 for 1 h at room temperature. An anti-mouse IgG was used as a primary antibody on one of the lungs as a control of a specificity of the method. For the immunolabelling visualization, Dako REAL EnVision Detection System (K5007, DAKO, Glostrup, Denmark) was used: the incubation with a peroxidase-conjugated polymer as a secondary antibody (for 30 min) was followed by the application of DAB+ Chromogen for a visualization of the reaction. The sections were counterstained with Mayer’s hematoxylin, dehydrated and mounted with cover slides. To assess viral antigen labelling, tissues were analyzed under a light microscope (Axiolab, Zeiss, Göttingen Germany).

### 2.7. Statistical Analysis

The genotype distribution of the SNPs was tested for deviation from the Hardy-Weinberg equilibrium (HWE) using the Court Lab HW calculator, including χ^2^ analysis (*p*-value < 0.05). The association of genotype frequencies with sheep’s SRLV status (positive and negative) was tested using the chi-square or Fisher’s exact test.

The relative risk (RR) to be SRLV-positive was estimated for animals carrying one and/or two copies of the putative susceptible allele (risk factor) with a method of Altman [30] using the following equation:

$$RR=\frac{a/(a+b)}{c/(c+d)}$$

where *a* is a number of SRLV-positive individuals carrying the risk factor, *b* is the number of SRLV-negative animals carrying the risk factor, *c* is the number of SRLV-positive individuals carrying no risk factor, and *d* is the number of SRLV-negative animals carrying no risk factor.

The *p*-value was calculated according to Sheskin [31] using MedCalc software [32]. The high RR value suggests a predisposition to the infection, and the risk and protective alleles were considered statistically significant with a *p*-value of <0.05.

A simple linear model approach was performed using a T-test to identify the association between genotypes and proviral loads. In this analysis, SNP was used as a classification variable, and proviral load values were used as analysis variables. Quantitative results as means ± SD presented significant differences between animals within given genotype groups.

## 3. Results

### 3.1. SRLV Status of Sheep

The presence of SRLV-specific antibodies was confirmed in 54 sheep (22 of the Polish Mountain breed, 21 of the Olkuska breed, and 11 of Cameroon sheep). At the same time, the remaining 53 were seronegative in the ELISA test. When DNA samples from all 107 sheep were tested by nested PCR, in 55 of them, 625 bp SRLV *gag* fragment was amplified, while the remaining 52 individuals were negative. Discordant results of PCR and ELISA were observed in five individuals, two of Cameroon and three of Olkuska breed. SRLV DNA was found in three animals, but no antibodies were detected. Such observations appeared in SRLV-infected sheep and were caused by poor antibody production, probably due to a low viral load or the early stage of infection. The opposite situation, characterized by the presence of an antibody without proviral DNA was noted in two sheep and can be explained by the low level of SRLV DNA. Therefore, to determine SRLV status of a particular animal, results of both tests were combined. Finally, 57 animals that were positive in at least one of the tests were considered SRLV-positive, while 50 animals that were negative in both ELISA and PCR tests were classified as SRLV-negative. Such an approach finally gave 22 SRLV-positive sheep of the Polish Mountain breed (42%), 23 of Olkuska (62%), and 12 of the Cameroon breeds (66.7%). These counted 53.3% SRLV-positive and 46,7% SRLV-negative individuals. The health status of the animals also varied. Among sheep from three flocks, eight animals with severe maedi-visna symptoms were noted, but most of the sheep represented healthy status.

### 3.2. Histopathology and Immunohistochemistry

Histopathological examination showed the microscopic lesions found in the lungs of the first sheep (Cameroon breed) included diffuse and extensive expansion of alveolar septa by hypertrophic smooth muscle cells, fibrosis, scattered macrophages, and lymphoid cells which shrank the alveolar and bronchiolar lumena. The lymphoid cells multifocally formed prominent lymphoid nodules (Figure S1A). These lesions were consistent with interstitial, severe, chronic, and diffuse interstitial pneumonia (ovine progressive interstitial pneumonia (OPP), also known as maedi disease). Furthermore, positive immunolabelling for the viral antigen was visible in specific macrophages and epithelial cells in the bronchi of this sheep (Figure S1B). In the lungs of the second sheep (Olkuska breed), multifocally visible areas of the interstitial infiltration of the alveolar septa by macrophages and fibrin, with occasionally present mild lymphohistiocytic perivascular cuffings, were observed (Figure S1C). The lesions indicated moderate chronic interstitial lymphohistiocytic pneumonia.

### 3.3. SNP Detection Within TMEM154 Locus

The Sanger sequencing allowed us to analyzed the total variability in exons 1 and 2 of TMEM154 gene in 107 sheep representing three breeds. The polymorphism identification showed the presence of 10 mutations, from which 8 were annotated in EVA (release 5, EMBL-EBI), and two were identified for the first time (Table 2). Most detected polymorphisms showed a high potential to impact gene expression (5′UTR variants) or protein sequence and function (frameshift and missense variants). Two novel polymorphisms were identified as missense and synonymous variants.

### 3.4. Genotype and Diplotype Analyses

Most of the detected SNPs were consistent with the HW equilibrium, but in some particular groups with a low number of individuals (<5), the HWE test was inaccurate (Table S2).

The genotype analysis showed that some polymorphisms were breed-specific, like missense variant rs427737740 (A/T, N70I) and rs429882112 (C/T, D33M), as well as both newly detected C > T polymorphisms; however, the frequency of the alternate allele in the last three polymorphic sites was shallow in the whole population. The following polymorphisms were detected only in two tested breeds (Polish Mountain sheep and Olkuska sheep), but not in Cameroon sheep: 5′UTR SNP (rs599267214 (C>T)), rs59493094 (C/del, R4AΔ53), and rs420489630 (C/T, T44M). Only two polymorphic sites were noted in all tested breeds. One of them, rs408593969 (G/A, E35K), showed interesting frequency. Alternate alleles were more frequent in the total population, and in Polish Mountain sheep and Olkuska breeds, it was equally represented in Cameroon sheep.

The association of genotype frequencies with SRLV status (positive and negative) of sheep was tested using the chi-square or Fisher’s exact test for most of the detected SNPs in all tested animals and particular breeds, but only if individuals carried both variants of each polymorphism). A significant association (*p* < 0.05) was found for 5′UTR SNP (rs599267214 (C>T)) only in the Olkuska breed, where all sheep carrying heterozygote of alternate homozygote were SRLV-negative. A strong association was also observed for rs420489630 (C/T, T44M) in Olkuska and Polish Mountain breeds and for 5′ UTR rs591381526 and rs408593969 in Cameroon sheep.

Genetic variations that generated amino acid substitutions gave 27 diplotypes with various numbers representing animals. Most of the diplotypes were represented by a low number of individuals (1–7) animals, but three showed higher frequencies: 25 (23.4%), 19 (17.8%), and 12 (11. 2%) (Table S3). The highest number of diplotypes was identified in Polish Mountain sheep (21 diplotypes) and the lowest number was observed in Cameroon Sheep (6 diplotypes). Strikingly, some diplotypes were noted only in SRLV-positive individuals and the other in SRLV-negative sheep. However, this observation was mainly related to less frequent diplotypes. There were also diplotypes with an equal number of SRLV-positive and -negative animals and higher rates of SRLV-infected animals. Interestingly, sheep with maedi-visna clinical symptoms represented various diplotypes (5, 12, 17, 22, and 24).

### 3.5. Relative Risk

Relative risk to be SRLV-positive was calculated based on the SRLV status of animals and genotypes containing one or two copies of putative risk alleles compared to those carrying only protective ones (Table 3). The relative risk was calculated separately for all animals as well as for animals of particular breeds. The highest RR scores in the total population and specific breeds were observed for genotypes GG and AG, encoding EE and KE at position 35, respectively; thus, they may be considered risk genotypes of TMEM154. However, they were statistically significant only for the whole population and the Olkuska breed (*p*-value: 0.004 and 0.005, respectively). In contrast, for Cameroon sheep with a relative risk of 13.46, the *p*-value slightly missed the significant threshold (0.057). The risk alleles might also be suggested CT and TT at position 14 of cDNA in 5′UTR SNP rs599267214, but the RR score was significant only in the Olkuska breed (RR = 3; *p*-value = 0.004). Similarly, a high score of relative risk was shown for alleles CG and GG in the other polymorphic site in 5′ UTR rs591381526 in the whole tested group (R = 1.25) and Cameroon sheep (R = 3), but only the last result was significant. Finally, genotypes CC at position 4 (R4A∆), 44 (T44M), and AA at position 70 (N70I) showed high relative risk results but without significance (*p*-value > 0.05).

### 3.6. Association Between SNPs and Provirus Copy Number

**Figure 1:**
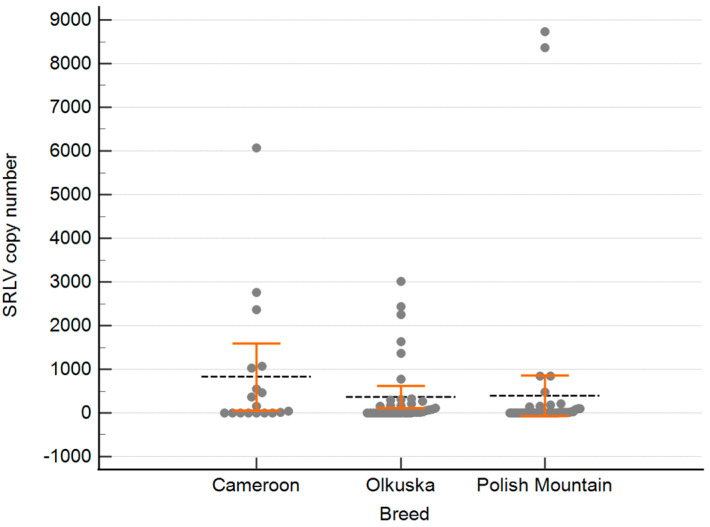
Distribution of the SRLV copy numbers in sheep from three breeds. The grey dots present the provirus copy number of each sheep, the black dashed line represents the mean of the SRLV copy number in each breed, and the orange error bars represent the 95% CI for the mean.

The level of SRLV proviral DNA was determined in blood leukocytes obtained from all PCR-positive sheep. The provirus copy number ranged between 6 and 8730 (mean: 877.4; median: 159.5) copies per 500 ng of genomic DNA. The distribution of provirus levels in sheep from particular breeds is shown in Figure 1.

**Figure 2:**
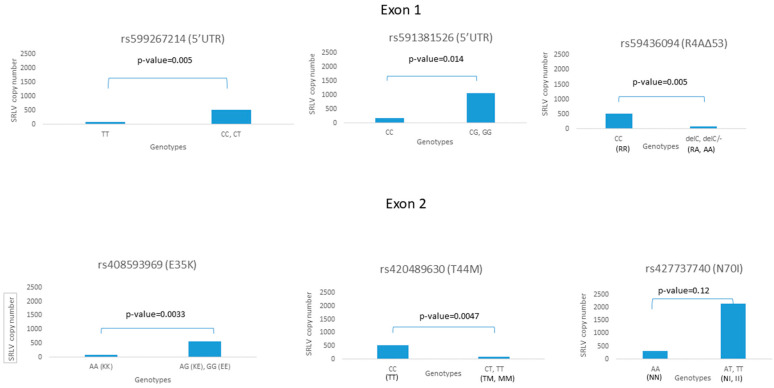
The association of TMEM154 genotypes with SRLV proviral load.

The association analysis showed statistically significant results between the average provirus copy number and five SNPs, where the proviral loads found in animals carrying tested alleles were analyzed against the proviral load of the remaining animals (Figure 2). Only SNPs with an allele frequency of at least 4% were analyzed.

The strongest association between genotype and proviral load was observed for polymorphic site rs408593969 (E35K) (*p* = 0.0033), where a significantly lower proviral load was observed in alternate homozygotes (AA, KK). Interestingly, all sheep of the Cameroon breed carrying this genotype were SRLV-negative. A similar observation was made for 5′UTR polymorphic site rs599267214 (*p* = 0.005), where animals with alternate homozygote TT showed a lower proviral load. A strong association was also noted for the second 5′UTR SNP (rs591381526) (*p* = 0.014), but in the opposite direction here, the reference (wild type) homozygote (CC) was strongly associated with a lower proviral load. A similar trend was observed for rs427737740, but this association was not statistically significant.

An interesting association was observed for rs594936094 (*p* = 0.005) and rs420489630 (*p* = 0.0047), where the heterozygotes and alternate homozygotes were linked with a lower proviral load.

**Figure 3:**
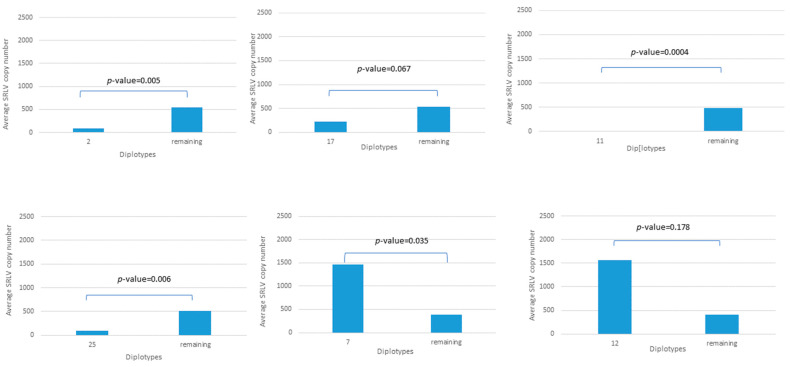
The association of selected TMEM154 diplotypes (according to Table S3) with SRLV proviral load.

When the relationship between diplotypes and mean SRLV proviral load was considered, the statistically significant association was noted for diplotypes 2, 7, 11, and 25 (according to Table S3) (Figure 3). The robust association was observed for diplotype 2 (*p*-value = 0.005) where only 16% animals were SRLV-positive, but with a very low SRLV proviral load, and for diplotype 11 carried by five SRLV-negative animals (*p*-value = 0.0004). An interesting group seemed to be sheep carrying diplotype 25, where 50% of animals were SRLV-positive but with a low proviral load, which resulted in a significant association. A similar trend was noted in sheep carrying diplotype 17 (25 individuals with diplotype 17), where although 76% were SRLV-positive, the mean proviral load was much lower than the rest of the animals but without significance (*p*-value = 0.067). All sheep with diplotypes 7 and 12 were seropositive, but a significant association with higher proviral load was observed only for diplotype 7 (*p*-value = 0.035).

### 3.7. Identification of SRLV Gag Subgroups

Nested PCR 625 bp fragments amplified from 28 SRLV-positive animals (5 Olkuska sheep, 12 Cameroon sheep, and 12 Polish Mountain sheep) were cloned. Between 1 and 5 sequences were generated for each animal and sequenced in both directions.

The phylogenetic analysis (Figure S2) showed that all six sequences derived from Olkuska sheep clustered with sequences of A18 SRLV genotype. Twelve sequences of Cameroon sheep were clustered together, and they were closely related to A5 genotype and British SRLV, isolate EV-1. Interestingly, 11 sequences obtained from Polish Mountain sheep were found in the A group in three distant clusters. Six sequences were closely related to A12 subgenotype, four were close to A17, and one sequence was identified within A24 subgenotype. The remaining twelfth sequence derived from Polish Mountain sheep (s_011/11_PM) clustered in B2 group, but with low bootstrap support.

## 4. Discussion

Since Heaton and co-workers [14] identified several polymorphisms in TMEM154 gene associated with the susceptibility of sheep to SRLV infection, similar studies were performed in different parts of the world on various breeds of sheep. However, such studies were not yet performed in regard to sheep from Poland. Therefore, we selected animals representing three breeds, including two native ones (Olkuska and Polish Mountain). All animals with SRLV-confirmed status were tested for the mutations in TMEM154 gene. The frequencies of the known and novel SNPs, as well as relative risk scores, were determined. Our study showed that sheep being GG homozygotes and heterozygotes AG, encoding EE or KE, respectively, at position 35 of the extracellular part of TMEM154 protein were more likely to be infected with SRLV. In turn, AA homozygotes animals, encoding KK isoform, seemed to be more resistant to infection. Such findings align with studies performed on different breeds from sheep populations in the USA, UK, Germany, Iran, Italy, Turkey, and Spain [14,17,19,23,33,34]. Some authors were cautious about the utility of this SNP as a marker of susceptibility due to significant results only for a fraction of tested breeds [17,34], as well as the fact that in some flocks, quite a high number of individuals carrying putative resistant variant are seropositive for SRLV [18,19]. Our study showed significant results for the whole tested animal population. However, when the analysis was performed concerning the particular breeds, relative risk scores were high; only the result of Olkuska sheep met the required statistical criterion of *p*-value of < 0.05. Surprisingly, all SRLV-infected animals in Cameroon sheep carried susceptible genotypes, while all SRLV-negative individuals carried only protective one. Furthermore, sheep carrying only the protective variant showed a significantly lower SRLV proviral load than the rest of the tested population carrying at least one copy of the susceptible variant. This finding may explain other authors’ concerns since a low proviral load has been previously associated with the limited spread of SRLV and lower odds of maedi-visna development [8,13]. Thus, it fits into the hypothesis of resistance. Alshanbari and co-workers [35] also observed a lower proviral load in ewes carrying protective genotype, but additionally, they found that the older the ewes, the fewer that carried one or two copies of the susceptible variant. Of course, this may be due to culling or premature death, but this finding is still very promising for the usefulness of E35K SNP in the control of SRLV infection.

Another SNP found by Heaton and co-workers is rs594936094, located in exon 1 [14]; it is related to the C deletion at codon position 4, resulting in frameshift mutation (R4A∆) in TMEM154 and truncation of the protein. Alternate homozygotes do not have functional TMEM154 protein. However, the animals carrying deleted variants remained healthy and produced healthy progeny [24]. Therefore, it may suggest that TMEM154 is not indispensable for sheep physiological functions. The considerable advantage of possessing this genotype is the high resistance to SRLV infection reported by some authors [14,24]. However, there are also cases of SRLV infection even in alternate homozygotes, but as reported by Clawson and co-workers, this ability is evidenced for specific lentivirus subgroups [24,36]. In our study, this SNP was observed only in native Polish breeds. Only one alternate homozygote was present in Olkuska sheep, which was indeed SRLV-negative. However, there were also eight heterozygotes in the Olkuska breed and five in Polish Mountain sheep, of which only four were SRLV-positive. In addition, a significantly lower SRLV proviral load was noted in heterozygotes and alternate homozygote, but a relative risk score, probably due to the low frequency, was beyond statistical significance. Freking and co-workers reported that ewes with this polymorphism do not differ from other animals in the flocks in terms of growth and reproduction; however, since the physiological function of TMEM154 has not been revealed yet, mating sheep carrying this SNP to reduce flock susceptibility to SRLV infection is not recommend [24]. Interestingly, Heaton and co-workers noticed that the deletion frameshift variant of R4A∆ is always present with T variant of SNP rs420489630 (C/T > T44M) located in exon 2 of TMEM154, thus suggesting a protective role or this pair of SNPs [14]. This finding was observed in all 14 Polish sheep carrying R4A∆ variant. Additionally, alternate homozygotes of both SNPs were noted together in one sheep, and heterozygotes came along in the remaining 13 sheep. Only one sheep of the Polish Mountain breed carried one copy of the alternate allele of T44M but without the deletion variant, and it was SRLV-positive. Frequent co-existence of both SNPs was related to a strong association with a lower proviral load in carrier sheep compared to in the rest of the tested population, thus strengthening its possible role as an SRLV-resistance marker. Polymorphism at position 70 of TMEM154 protein (rs427737740, A/T, N70I) was observed only in a fraction of Polish Mountain sheep (eight animals), of which only three were SRLV-positive. It was previously reported in US and Italian sheep as a potential SRLV risk factor [16,22], but in our study, the results were contrary and insignificant. However, it is worth mentioning that two sheep carrying alternate homozygotes (TT > II) and heterozygotes (AT > NI), respectively, showed the presence of a highest proviral load (both over 8000 copies) observed in the tested population.

The interesting site polymorphisms are those observed in the 5′UTR region of TMEM154. Based on the relative risk scores and association with a proviral load, we can only speculate that alternate homozygotes and heterozygotes (CT and TT) of rs599267214 at position 14 and of rs591381526 (CG and GG) may be related to susceptibility to SRLV infection. Untranslated regions (UTRs) are the part of genes flanking the protein-coding sequence that are included in the mRNA but are not translated into protein. They are considered essential mediators of post-transcriptional regulation since they include regulatory elements and, therefore, control mRNA stability, the rate of protein synthesis, and its cellular localization [37,38]. UTRs vary across genes, in size, and in the composition of regulatory elements responsible for binding to various proteins and non-coding RNAs. These SNPs were not previously described in the context of SRLV infection or TMEM154 regulation; thus, their role remains unknown.

Moretti and co-workers [19] suggested a potential protective role of E35K polymorphism in TMEM154 gene regarding infection with genotype A SRLV, but not with genotype B or E. Since this study was performed in three Italian breeds, it was interesting whether such a correlation could be found in Polish flocks. Molecular characterization of the SRLV *gag* was performed for all infected Cameroon sheep, most of the infected Olkuska sheep, and a fraction of Polish Mountain sheep. We have observed circulation of different but homogenous SRLV subtypes of genotype A in both Olkuska (A18) and Cameroon breeds (closely related to A5 and Scotish EV-1 strain) and a couple of distant A subtypes and one B genotype in Polish Mountain sheep. Due to the low number of animals infected with the SRLV B genotype and several (mixed) genotypes noted in each breed, the association was not analyzed. The sheep demonstrating maedi-visna symptoms were infected with different SRLV subtypes, representing different genotypes. Therefore, no relationship between the severity of maedi-visna symptoms and particular SNPs was explored in our study.

## 5. Conclusions

This paper presents the first report on the prevalence of polymorphisms in the TMEM154 gene in sheep from Poland and their association with SRLV infection status and proviral load. Our findings align with previous studies, since E35K and R4A∆/T44M SNPs, recorded during our surveys, were associated with susceptibility to SRLV infection and proviral load. They suggest that selecting SRLV-resistant animals based on identification of particular polymorphisms of TMEM154 gene would be a promising way to control SRLV infection, but further validation in a larger group of sheep is required.

## Figures and Tables

**Table 1:** Primers used for the amplification of fragments from two exons of TMEM154 gene.

TMEM154 Gene	Primers Used *	Amplicon Length	Reference Number
Exon 1	5′-GCGAGGCGTGCTAACTG-3′	589 bp	ENSOARE00020168597
	5′-GCCCATTAAAGCCGGT-3′		
Exon 2	5′-GAGGGTAAGTTTCAGATCATTG-3′	554 bp	ENSOARE00020168636
	5′-TTATGTAGCTGCTTCACTTAAA-3′		

* According to Arcangeli et al. [22].

**Table 2:** Single nucleotide polymorphisms found in TMEM154 gene sheep from three breeds.

SNP	HGVS Nomenclature	Accession Number	Type of Modification	Localization
C/T	ENSOART00020031155.2:c.-15C>T	rs599267214	5 prime UTR variant	Exon 1
G/C	ENSOART00020031155.2:c.-1G>C	rs591381526	5 prime UTR variant	Exon 1
C/-	ENSOARP00020025739.2:p.Arg4AlafsTer53 (R4A^Δ^)	rs594936094	Frameshift variant	Exon 1
C/T	ENSOARP00020025739.2:p.Thr25Ile(T25I)	novel	Missense variant	Exon 1
G/A	ENSOARP00020025739.2:p.Asp33Asn (D33N)	rs429882112	Missense variant	Exon 2
G/A	ENSOARP00020025739.2:p.Glu35Lys (E35K)	rs408593969	Missense variant	Exon 2
G/C	ENSOARP00020025739.2:p.Gly38Arg (G38R)	rs1088921014	Missense variant	Exon 2
C/T	ENSOARP00020025739.2:c.C117T	novel	Synonymous variant	Exon 2
C/T	ENSOARP00020025739.2:p.Thr44Met (T44M)	rs420489630	Missense variant	Exon 2
A/T	ENSOARP00020025739.2:p.Asn70Ile (N70I)	rs427737740	Missense variant	Exon 2

**Table 3:** Relative risk of infection of SRLV infection for SNPs in TMEM154 gene. *, *p*-value ≤ 0.05; the score is statistically significant.

SNP	Genotypes	Total	Breed		
			Polish Mountain Sheep	Olkuska Sheep	Cameroon Sheep
		Relative Risk (95% CI)			
rs599267214 (C/T) 5′UTR	CC vs. CT, TT	0.74 (0.492–1.1004)	0.47 (0.17–1.34)	0.33 (0.18–0.61)	1.316 (0.18–9.59)
	CT, TT vs. CC	1.357 (0.91–2.03)	2.11 (0.74–5.99)	3 (1.64–5.49)	0.76 (0.10–5.54)
	*p*-value	0.135	0.16	0.004 *	0.79
rs591381526 (C/G) 5′UTR	CC vs. CG, GG	0.80 (0.56–1.13)	1.02 (0.48–2.18)	1.24 (0.69–2.20)	0.33 (0.13–0.84)
	CG, GG vs. CC	1.25 (0.88–1.78)	0.98 (0.46–2.10)	0.81 (0.45–1.44)	3 (1.19–7.56)
	*p*-value	0.21	0.96	0.47	0.019 *
rs5943094 CC/del R4AΔ	CC vs. C/-, -/-	1.99 (0.85–4.65)	2.23 (0.38–13.27)	2.14 (0.83–5.56)	na
	C/-, -/- vs. CC	0.5 (0.21–1.17)	0.47 (0.07–2.66)	0.47 (0.18–1.21)	na
	*p*-value	0.11	0.38	0.11	na
rs408593969 (G/A) E35K	AA vs. AG, GG	0.26 (0.10–0.65)	0.15 (0.01–2.19)	0.44 (0.19–1.00)	0.07 (0.005–1.08)
	AG, GG vs. AA	3.8 (1.54–9.51)	6.7 (0.46–98.50)	2.28 (0.99–5.29)	13.46 (0.93–195.01)
	*p*-value	0.004 *	0.16	0.05 *	0.057
rs420489630 (C/T) T44M	CC vs. CT TT	1.69 (0.81–3.55)	1.30 (0.40–4.24)	2.14 (0.83–5.56)	na
	CT, TT vs. CC	0.59 (0.28–1.23)	0.76 (0.24–2.49)	0.467 (0.18–1.21)	na
	*p*-value	0.16	0.66	0.117	na
rs427737740 (A/T) N70I	AA vs. AT, TT	1.45 (0.58–3.62)	1.15 (0.44–2.99)	na	na
	AT, TT vs. AA	0.69 (0.27–1.71)	0.87 (0.33–2.26)	na	na
	*p*-value	0.42	0.77	na	na

na—not applicable due to lack of sheep carrying the alternative allele.

## Data Availability

Data supporting reported results are available in this article and in the Supplementary Materials.

## Supplementary Materials

The following supporting information can be downloaded at: https://www.mdpi.com/article/10.3390/pathogens14010016/s1, Figure S1: Example photomicrographs of lung sections from the SRLV-infected sheep; Figure S2: Phylogenetic tree inferred from the 393 bp sequence of SRLV gag region amplified from 28 sheep and extracted from partial and complete *gag* sequences of SRLV available in GenBank; Table S1: Primers and probes used in qPCR assays; Table S2: The frequencies of alleles and genotypes noted in TMEM154 of sheep from three breeds; TableS3: The frequencies of diplotypes noted in TMEM154 of sheep from three breeds.
