# Supplementary material for: First Report of SNPs Detection in TMEM154 Gene in Sheep from Poland and Their Association with SRLV Infection Status
Source: Pathogens. 2024 Dec 30;14(1):16. doi: 10.3390/pathogens14010016 (PMC11768335; doi:10.3390/pathogens14010016)
Supplement: Supplementary file 1 [file pathogens-14-00016-s001.zip › pathogens-3384153-supplementary.pdf]

Table S1. Primers and probes used in qPCR assays.

Primer	Primer Sequence	Amplicon Length
qCAgag5	GCrGGrGGGAAGrAGyTGGAA	103 bp
qCAgag3	TCCTCrGACACrAGkCCATGyTGC	
qCAgag5_OL	CGGGAGGGAAGGAGTTGGAA	105 bp
qCAgag3_OL	CAGCTGTCTCTCAAAGTCCTCG	
qCAgag_P_OL	6-FAM-GTAGAGTCAGTGGTCTTCCAGCAGCT-BHQ	
CAgag16_Fw	AGCAATGCAGCATGGACTTGTGT	120 bp
CAgag16_Rev	TGTGCTCTGTTCCCAGGCATCAT	
CAgag16_P	6-FAM CTGAGGATTTTGAGAGGCAATTAGCCT-BHQ-1	

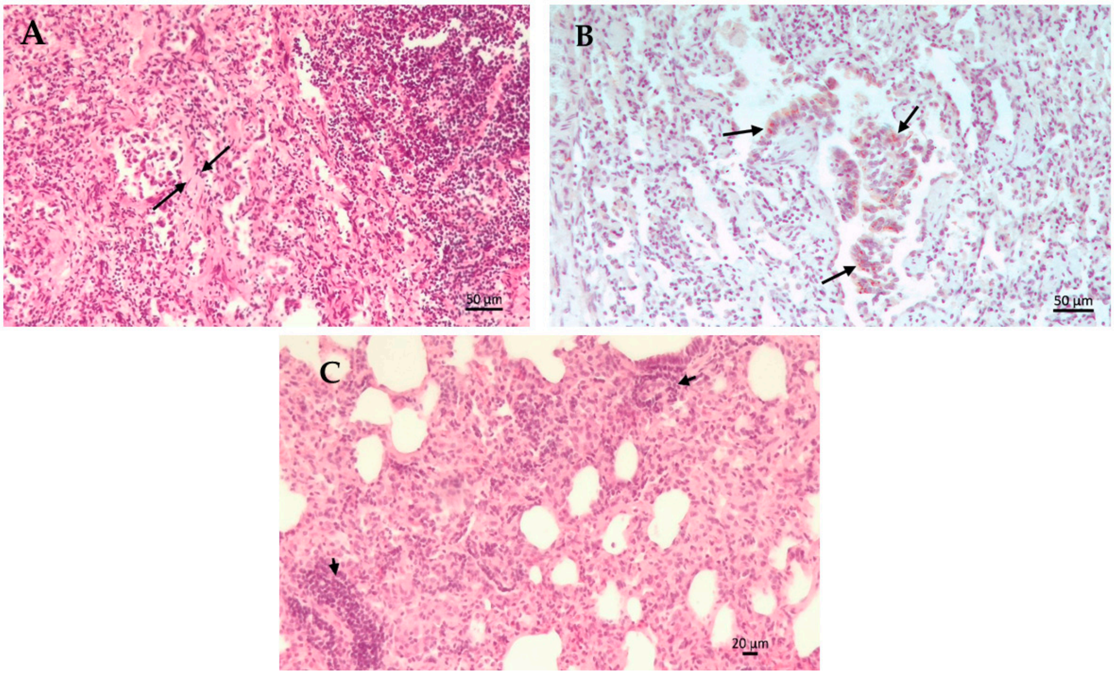

Figure S1. Example photomicrographs of lung sections from the SRLV-infected sheep. (A) Lung tissue from the Cameroon sheep, stained with hematoxylin and eosin (HE). Visible are the alveolar septa thickened by fibrosis (arrows), diffuse infiltration of the alveoli with lymphocytes and macrophages and, on the right side, an aggregation of lymphocytes forming a nodule. (B) Immunohistochemical (IHC) staining of the lung tissue from the Cameroon sheep for specific SRLV p28 detection. The positive reaction is visible as the brown staining in the bronchiolar epithelial cells and singular macrophages (arrows). (C) Lung tissue from the Olkuska sheep, stained with hematoxylin and eosin (HE). There is an expansion of alveolar septa by fibrin and macrophages infiltrating the interstitium, accompanied by moderate lymhocytic perivascular cuffings (arrows).

Table S2. The frequencies of alleles and genotypes noted in TMEM154 of sheep from three breeds. HWE – Hardy-Weinberg Equilibrium. If p-value < 0.05 - not consistent with HWE. Not accurate if <5 individuals in any genotype group; ref – reference allele/homozygote, het – heterozygote, alt – alternate allele/homozygote.

SNP Symbol and localization		Genotype/Allele		FREQUENCIES (number of sheep)			
				Total	Breed		
					Polish Mountain sheep	Olkuska Sheep	Cameroon Sheep
EXON 1	rs599267214 (C/T) (5' UTR)	Genotypes	CC ref	0.486 (53)	0.25 (13)	0.595 (22)	1 (18)
			CT het	0.374 (40)	0.48 (25)	0.405 (15)	0 (0)
			TT alt	0.14 (14)	0.27 (14)	0 (0)	0(0)
		Allels	C ref	0.675	0.49	0.775	1
			T alt	0.325	0.51	0.225	0
			HWE (p-value)	0.154	0.783	0.122	NA
	rs591381526 (C/G) (5' UTR)	Genotypes	CC ref	0.682 (73)	0.769 (40)	0.649 (24)	0.50 (9)
			CG het	0.243(26)	0.211 (11)	0.27 (10)	0.278 (5)
			GG alt	0.075 (8)	0.019 (1)	0.081 (3)	0.222 (4)
		Alleles	C	0.8	0.875	0.785	0.64
			G	0.2	0.125	0.215	0.36
			HWE (p-value)	0.017	0.812	0.218	0.09
	rs59493094 (C/del) (R4A$^{\Delta 53}$)	Genotypes	CC ref	0.87 (93)	0.904 (47)	0.757 (28)	1 (18)
			C/- het	0.121 (13)	0.096 (5)	0.216 (8)	0 (0)
			-/- alt	0.009 (1)	0 (0)	0.027 (1)	0 (0)
		Alleles	C ref	0.93	0.95	0.87	1
			- alt	0.07	0.05	0.13	0
			HWE (p-value)	0.482	0.716	0.648	NA
	novel C --> T (T25I)	Genotypes	CC ref	0.981 (105)	0.96 (50)	1 (37)	1 (18)
			CT het	0.019 (2)	0.04 (2)	0 (0)	0 (0)
			TT alt	0 (0)	0 (0)	0 (0)	0 (0)
		Alleles	C ref	0.99	0.98	1	1
			T alt	0.01	0.02	0	0
			HWE (p-value)	0.922	0.89	NA	NA
EXON2	rs429882112 (C/T) (D33N)	Genotypes	CC ref	0.991 (106)	0.98 (51)	1 (37)	1 (18)
			CT het	0.009 (1)	0.02 (1)	0 (0)	0 (0)
			TT alt	0 (0)	0 (0)	0 (0)	0 (0)
		Alleles	C ref	0.99	0.99	1	1
			T alt	0.01	0.01	0	0
			HWE (p-value)	0.96	0.944	NA	NA
	rs408593969 (G/A) (E35K)	Genotypes	GG ref	0.336 (36)	0.44 (23)	0.190 (7)	0.33 (6)
			AG het	0.44 (47)	0.44 (23)	0.486 (18)	0.33 (6)
			AA alt	0.224 (24)	0.12 6)	0.324 (12)	0.33 (6)
		Alleles	G ref	0.44	0.34	0.43	0.5
			A alt	0.56	0.66	00.57	0.5
			HWE (p-value)	0.254	0.945	0.957	0.16
	C-- >T nov	Genotypes	CC ref	0.98 (105)	0.96 (50)	1 (37)	1 (18)

		CT het	0.02 (2)	0.04 (2)	0 (0)	0 (0)
		TT alt	0 (0)	0 (0)	0 (0)	0 (0)
	Alleles	C ref	0.99	0.97	1	1
		T alt	0.01	0.03	0	0
		HWE p-value	0.92	0.887	NA	NA
rs420489630 (C/T) (T44M)	Genotypes	CC ref	0.86 (92)	0.88 (46)	0.76 (28)	1 (18)
		CT het	0.13 (14)	0.12 (6)	0.21 (8)	0 (0)
		TT alt	0.01 (1)	0 (0)	0.03 (1)	0 (0)
	Alleles	C ref	0.93	0.94	0.87	1
		T alt	0.07	0.06	0.13	0
		HWE (p-value)	0.53	0.52	0.31	NA
rs427737740 (A/T) (N70I)	Genotypes	AA ref	0.92 (99)	0.85 (44)	1 (37)	1 (18)
		AT het	0.07 (7)	0.13 (7)	0 (0)	0 (0)
		TT alt	0.01 (1)	0.02 (1)	0 (0)	0 (00
	Alleles	A ref	0.96	0.92	1	1
		T alt	0.04	0.08	0	0
		HWE (p-value)	0.05	0.28	NA	NA

Table S3. The frequencies of diplotypes noted in TMEM154 of sheep from three breeds.

#	**Diplotype** **Position of affected codon in TMEM 154**										no.	pop freq (107)	PM ferq (52)	C freq (18)	Olk freq (37)	SRLV Positive %
	**5′UTR**		**4**	**25**	**33**	**35**	**38**	**117**	**44**	**70**						
1	CC	CC	CC	CC	GA	AG	GG	CC	CC	AA	1	0.009	0.019	0	0	0
2	CC	CC	CC	CC	GG	AA	GG	CC	CC	AA	19	0.18	0.038	0.33	0.3	16
3	CC	CC	CC	CC	GG	AG	GG	CC	CC	AA	2	0.019	0	0.11	0	100
4	CC	CC	CC	CC	GG	AG	GG	CC	CC	AT	3	0.028	0.058	0	0	33
5	CC	CC	CC	CC	GG	GG	GG	CC	CC	AA	2	0.019	0.019	0.056	0	100
6	CC	CG	CC	CC	GG	AA	GG	CC	CC	AA	2	0.019	0.038	0	0	0
7	CC	CG	CC	CC	GG	AG	GG	CC	CC	AA	7	0.065	0	0.222	0.081	100
8	CC	CG	CC	CC	GG	GG	GG	CC	CC	AA	1	0,009	0	0.056	0	100
9	CC	CG	CC	CC	GG	GG	GG	CC	CC	TT	1	0.009	0.019	0	0	100
10	CC	CG	CC	CT	GG	AA	GG	CC	CC	AA	1	0.009	0.019	0	0	0
11	CC	CG	C/-	CC	GG	AG	GG	CC	CT	AA	5	0.047	0.019	0	0.11	0
12	CC	GG	CC	CC	GG	GG	GG	CC	CC	AA	5	0.047	0	0.22	0.027	100
13	CC	GG	del/del	CC	GG	GG	GG	CC	TT	AA	1	0.009	0	0	0.027	0
14	CC	GG	C/del	CC	GG	GG	GG	CC	CT	AA	1	0.009	0	0	0.027	0
15	CC	GG	C/del	CT	GG	AG	GG	CC	CT	AA	1	0.009	0.019	0	0	0
16	CT	CC	CC	CC	GG	AA	GG	CC	CC	AA	2	0.019	0.019	0	0.027	50
17	CT	CC	CC	CC	GG	AG	GG	CC	CC	AA	25	0.234	0.269	0	0.297	76
18	CT	CC	CC	CC	GG	GG	GG	CC	CC	AA	1	0.009	0.019	0	0	0
19	CT	CC	CC	CC	GG	GG	GG	CC	CC	AT	3	0.028	0.058	0	0	0
20	CT	CG	CC	CC	GG	AG	GG	CC	CC	AA	1	0.009	0.019	0	0	100
21	CT	CG	CC	CC	GG	AG	GG	CT	CC	AA	1	0.009	0.019	0	0	100
22	CT	CG	CC	CC	GG	GG	GG	CC	CC	AT	1	0.009	0.019	0	0	100
23	CT	CG	C/del	CC	GG	GG	GG	CC	CT	AA	6	0.056	0.058	0	0.081	66.7
24	TT	CC	CC	CC	GG	AG	GG	CC	CC	AA	1	0.009	0.019	0	0	100
25	TT	CC	CC	CC	GG	GG	GG	CC	CC	AA	12	0.112	0.212	0	0.027	50
26	TT	CC	CC	CC	GG	GG	GG	CC	CT	AA	1	0.009	0.019	0	0	100
27	TT	CC	CC	CC	GG	GG	GG	CT	CC	AA	1	0.009	0.019	0	0	0

pop freq - frequency for the whole population, PM - Polish Mountain sheep, Olk - Olkuska sheep, C- Cameroon sheep, del - deletion

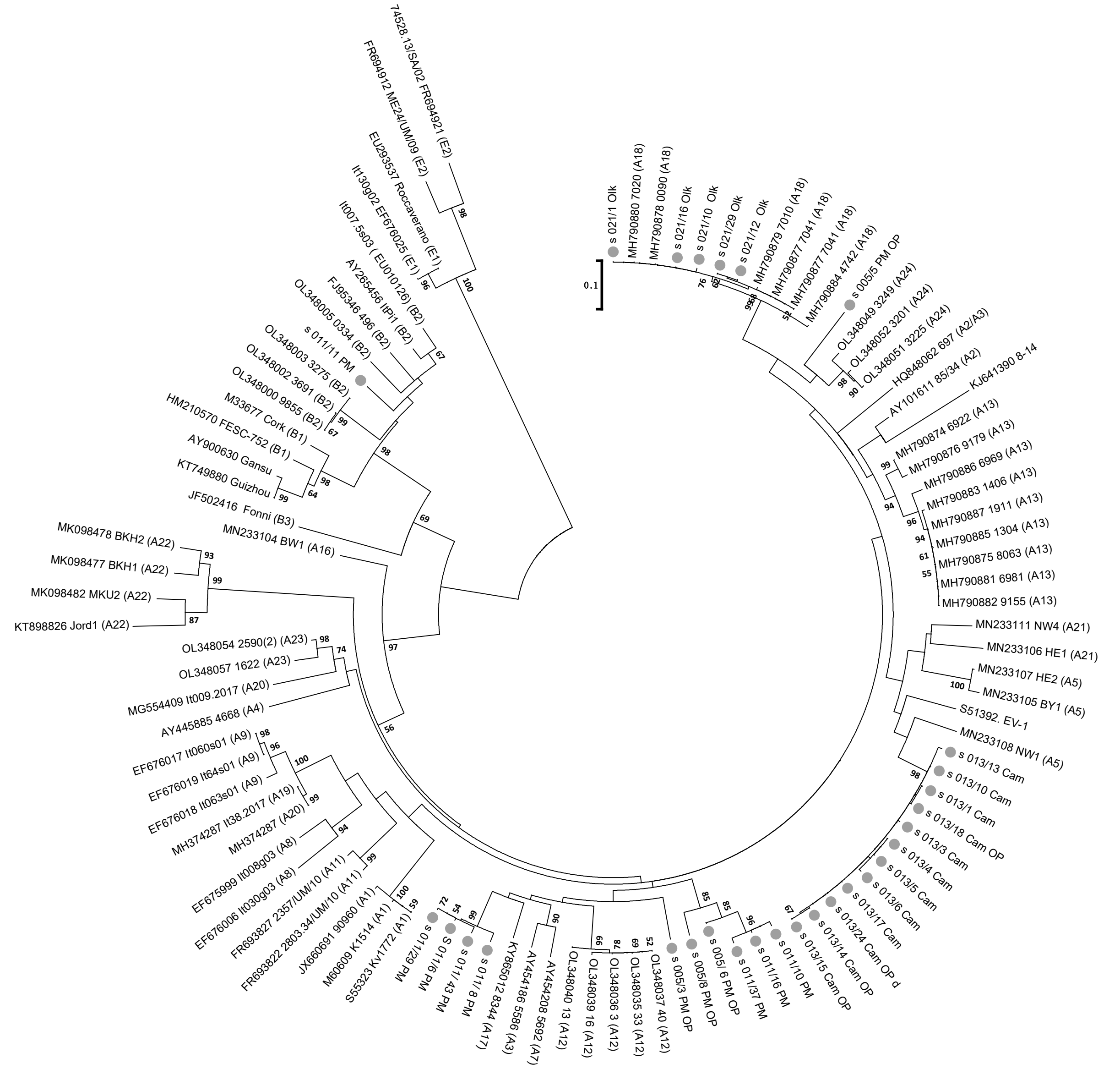

Figure S2. Phylogenetic tree inferred from the 393 bp sequence of SRLV *gag* region amplified from 28 sheep and extracted from partial and complete *gag* sequences of SRLV available in GenBank (accession numbers are shown on the tree). All analyzed sequences were devoid of primer sequences. The analyses were conducted in MEGA 6 using the Maximum Likelihood method and Tamura 3-parameter model with the gamma distribution (+G) and evolutionarily invariable sites(+*I*) with five rate categories. Numbers at the branches indicate the percentage of bootstrap values obtained from 1000 replicates. The isolates from this study are reported with grey dots.
